# Anesthesiologists’ conceptions of learning anesthesia in the context of their specialty training program: a phenomenographic study

**DOI:** 10.1186/s12909-023-04573-x

**Published:** 2023-08-21

**Authors:** Hanna Chin, Åke Ingerman, Helena Odenstedt Hergès

**Affiliations:** 1 Department of Anaesthesia and Intensive Care, Region Västra Götaland, Sahlgrenska University Hospital, Gothenburg, Sweden; 2 Department of Anesthesiology, Institute of Clinical Sciences, Sahlgrenska Academy, University of Gothenburg, Gothenburg, Sweden; 3 Department of Pedagogical, Curricular and Professional Studies, University of Gothenburg, Gothenburg, Sweden

## Abstract

**Background:**

Training anesthesiologists poses challenges and complexities, particularly in defining and teaching excellence in anesthesia. Existing anesthesia curricula primarily emphasize the acquisition of knowledge, practical skills, and professional competencies, often neglecting the development of intangible skills like tacit knowledge. Despite efforts to establish learning goals through carefully describing competencies, there is a risk of oversimplifying the intricate aspects of professional anesthesia practice. Therefore, the objective of this study is to gain a deeper understanding of the genuine curriculum of a specialty training program in anesthesia. This will be achieved by exploring the perceptions of learners with different levels of experience within the program.

**Methods:**

This study employs a phenomenographic research approach to explore the conceptions of anesthesiology trainees and specialists, specifically from a student’s perspective, regarding what constitutes an excellent anesthesiologist i.e., what to learn, and the learning process associated with it.

**Results:**

This study identified three different conceptions of learning anesthesia within the context of a specialty training program: “Learning Competencies of Anesthesia,“ “Learning Work as an Anesthesiologist” and “Learning Being an Anesthesiology Professional.“ These conceptions ranged from a relatively instrumental view of education and self-responsibility for learning to a perspective of continuous personal reflection and development integrated with professional interaction. The three conceptions can be described in six dimensions describing the variation in approach to learning and the conceptualization of an anesthesiologist. Relationships between the conceptions and the dimensions were represented in a descriptive framework, showing the hierarchy of increasing understanding.

**Conclusion:**

This study has uncovered diverse learner perspectives among anesthesiologists at various experience levels concerning their understanding of the role of an anesthesiologist and the associated learning process. These distinct understandings can be categorized into different groups and presented in a descriptive framework that encapsulates the fundamental elements and important educational aspects of an anesthesiologist’s progression through a specialty training program in anesthesia. By recognizing and integrating these diverse perspectives, anesthesia education can be enhanced, ultimately resulting in improved preparation of future anesthesia curriculum, teaching and assessments.

## Background

Training to become an anesthesiologist is an advanced form of education that requires a high level of domain knowledge and adaptation to skilled professional practice. Furthermore, defining excellence in anesthesia and determining the best way to teach it to aspiring trainees is a challenging task in itself. The educational needs are complex and go beyond simple activities to ensure the knowledge, practical skills, and professional competencies outlined in current formal anesthesia curriculum documents [1, 2]. This reflects the tension between, on the one hand, the educational tradition in theoretically oriented university programs and upper secondary school in the domain of science, and on the other hand, the practical and socially integrated professional practice of anesthesiologists. The former is dominated by well-articulated knowledge structures, concepts, and facts, while the latter is characterized by complex capabilities and tacit knowledge, as well as collective and cross-professional communication and collaboration.

Many of the non-technical skills required of an anesthesiologist, such as clinical reasoning [3], communication [4], and context-specific knowledge application, rely heavily on the acquisition of tacit knowledge [5]. As such, it has been argued that learning tacit knowledge is vital for becoming an excellent anesthesiologist, rather than just a competent one [6]. However, because these competencies are difficult to describe, they are often overlooked when organizing specialty training programs in anesthesia. Instead, the focus tends to be on competencies that are more easily measurable, leading to a significant part of the true curriculum being missed [7].

As a result, anesthesia education often places heavy emphasis on the teaching and formal assessment of theoretical knowledge, coupled with a practice-based professional apprenticeship in which curricula and assessment thereof is not clearly defined. This has been identified as a problem, along with the lack of transparent processes for quality assurance in specialist education curricula. Recently, competency-based curricula and assessments have been proposed as a systematic approach to bridging the gap between these different traditions. It is also argued that this approach may provide clear quality control of student learning and a way to articulate competencies that can be formally assessed [8].

However, even with this approach, many complex aspects of professional practice remain to be described. This approach has also been criticized for attempting to reduce something that is fundamentally non-linear and complex into linear and separable competencies [9]. This is because the context of education to become a specialist, and the essential knowledge and capabilities required in anesthesia, are not readily available for study. As a result, the research field is dominated by the experiences of practitioners and expert perspectives [10].

The competency-based approach, formal education, and the professional perspective all imply a teacher or expert perspective on what it takes to develop excellence in anesthesia. However, there is a lack of studies that contribute to understanding what the education and profession entail from a student perspective. This is essential to capture the complex aspects of developing excellence in anesthesia. Such studies also require access to, and a nuanced understanding of, relevant contexts.

The importance of such studies is underscored by current efforts for quality assurance mentioned above, which run the risk of missing the taken-for-granted goal of a broader set of competencies outside the easily defined aspects of being an anesthesiologist.

This study aims to complement the available literature on anesthesia education to address these risks. Specifically, we aim to investigate how trainees and educational supervisors perceive learning anesthesia in the context of specialty training program. To address this aim, we have taken a phenomenographic research approach.

We describe the investigated phenomenon in terms of two aspects: what is perceived as constituting an excellent anesthesiologist (i.e., what learning is directed towards by the learner) and, how the learning process when immersed in a specialty training program in anesthesia is perceived (i.e., how the learning process as such is perceived by the learner). This will result in a well-articulated descriptive framework of the essence and the important educational aspects of a specialty training program in anesthesia.

## Method

### Phenomenography as a research approach

To understand intricate social phenomena, such as anesthesiology training in a hospital setting, employing a qualitative research method is advantageous, as suggested by Bould et al.[11]. This approach centers on investigating human experiences within a particular context, instead of establishing cause and effect relationships through statistical means. It is ideal for creating hypotheses rather than verifying them through prediction and testing. The study is thus exploratory in nature.

Phenomenography is a qualitative research approach that aims to reflect the qualitative differences in how people perceive, understand, or conceptualize phenomena in the world, rather than describing the phenomena themselves [12]. The relevant phenomena range from well-delimited disciplinary concepts, such as experiences of specific situations and tasks, for example, reading an anesthesiology textbook, to broader experiences, such as how learning is perceived in an anesthesiology specialist training program, as is the case in this study.

Phenomenographic results consist of a set of categories that are characterized by distinct ways of conceptualizing a phenomenon. These categories are logically and often hierarchically related. Phenomenographic studies use empirical data, often gathered through interviews with individuals, but the results do not reflect specific individuals. Instead, they reflect the same individual in different situations or different individuals who apparently understand the phenomenon differently, even within the same contextual or historical background. At the same time, this qualitative variation is limited, even though experience, in an ultimate sense, is inexhaustible.

One concern in any research study of this kind is the potential for interviewer bias. However, in phenomenographic studies, the focus is on understanding the variations in participants’ personal experiences and perspectives, rather than seeking a single essence or truth. Therefore, the interviewer’s opinions or biases would not fundamentally distort the participants’ interpretations and understandings – the participants cannot step outside their own repertoire of how they may understand the phenomenon. Individual participants may delimit what they share in an interview, but on the level of the group, different ways of understanding are normally present (and frequencies of different ways of understanding is not in focus).

Phenomenography as a research approach, and the results from such investigations, are particularly relevant in educational contexts [13, 14]. The way we perceive what we need to learn and how to learn it determines how we engage with educational activities [13, 14]. Similarly, the learning conceptions of educators influence their teaching practices [15]. Furthermore, identifying the variation in the conceptualization of a phenomenon can highlight the key elements that students struggle to learn.

### Data collection

The empirical data for this study was collected through semi-structured interviews of specialty trainees and qualified specialists with varying experience in anesthesia (see Table 1).

**Table 1:** Demographics of participants

	Total	Female	Male
Trainee year 1	6	3	3
Trainee year 2–3	4	2	2
Specialists	5	2	3

An interview guide was used consisting of a number of open questions (Table 2.) During the interview, follow-on questions then sought to clarify intended meanings behind the answers, which included asking for descriptions of concrete experiences. While asking the questions, the interviewer avoided to impose her own conceptions, leading questions or introducing new terms or correcting answers with own expressions. After each question enough time and space were given for the participant to adequately reflect and give an in-depth answer.

**Table 2:** Interview guide

Open questions:
• Describe the specialty trainee program to a doctor new to anesthesiology
• What is the difference between this postgraduate program and other educational programs you have experienced so far?
• If you could construct your own training program, what would it look like?
• Describe a day that was highly educational and a day that was not educational.
• Describe an excellent and a poor anesthesiologist.

Two pilot interviews were performed to ensure the phenomenon was clearly in focus in the interview and described across a range of aspects by the interviewees as well as hone the researcher’s interview skills. This data was also included in the analysis. Interviews were performed by the primary researcher (HC). Each interview lasted between 20 and 40 min and were audio-recorded with iPhone Voice Memos. The audio-recordings were transcribed verbatim by the primary researcher and field notes made of relevant contextual features of the interviews.

### Setting

The interviews were conducted at a university hospital in Sweden that offers a comprehensive anesthesiology and intensive care training program lasting a minimum of five years. This program comprises clinical rotations, mandatory courses, and dedicated time for self-directed study and research. All aspects of the program adhere to the guidelines set by the National Board of Health and Welfare in Sweden, as well as the recommendations established by the Swedish Association for Anesthesiology and Intensive Care. Upon completion of the program, graduates work as physicians specializing in both anesthesia and intensive care medicine.

### Participant selection

Phenomenography aims to capture the diversity of perspectives that individuals may have about a certain phenomenon. To achieve this goal, the study utilized purposive sampling, which involved selecting participants with a range of backgrounds, academic abilities, and experience in the field of anesthesia, to maximize the variation in their responses. After conducting 15 interviews, data saturation was achieved, meaning that little new or different insights were obtained from additional interviews. Therefore, it was concluded that further interviews would not yield any additional findings.

### Data analysis

The transcribed interview data was analyzed using the seven steps of analysis in phenomenography described by Dahlgren and Fallberg [16]. Table 3. shows the steps in this study.

**Table 3:** Seven steps of analysis in phenomenography for this study

Steps	Data analysis Dahlgren & Fallberg	Data analysis this study
1	Familiarization	All transcripts read by HC & S; 5 transcripts by HOH, ÅI
2	Condensation	Identifying and coding meaningful units in the transcripts: Coding of 5 transcripts by HC, HOH, ÅI. Then all transcripts by HC
3	Comparison	Comparing the units with similarities and differences: HC with group discussion HC, HOH, ÅI
4	Grouping	Allocating answers expressing similar ways of understanding the phenomenon to the same conceptions: HC with group discussion HC, HOH ÅI. External validation by external field experts.
5	Articulating	Capturing the essential meaning of a certain conception. HC & group discussion
6	Labelling	Expressing the core meaning of the conception. HC & group discussion
7	Contrasting	Comparison of conceptions with regard to similarities and differences. HC & group discussion

### Research team and reflexivity

The phenomenographic study adhered to rigorous standards of quality throughout its various stages, as recommended by Sin [17], and adhered to the COREQ checklist for reporting the findings. The research team and external experts consisted of individuals with a strong background in qualitative research, particularly in the field of phenomenography, as well as professionals with expertise in anesthesia postgraduate education.

Validation of the study was sought by presenting the comprehensive results to colleagues who work in the same context as the interviewees, aiming to receive their feedback and determine if the various ways of experiencing were recognized and deemed valuable as explanatory tools. Furthermore, the interview findings were regularly reviewed and discussed in team meetings. Through this process, a consensus was reached by triangulating the insights from both the core research team and external experts. The diverse backgrounds of the research members contributed to the robustness of this triangulation process.

Throughout the study, all research members actively engaged in reflective practices and critically examined their personal perspectives to minimize bias.

## Results

Three qualitatively different conceptions of learning anesthesia were found within the context of their specialty training program. These are described below under the names of *“Learning Competencies of Anesthesia,“ “Learning Work as an Anesthesiologist”* and *“Learning Being an Anesthesiology Professional.“* These conceptions reflect, on one end, a relatively instrumental view of both the education that they are engaged in and their own responsibility for learning, and on the other end, a perspective of continuous reflection and development integrated with professional interaction with other anesthesiologists as well as other medical professionals.

The three conceptions have undergone further analysis across six dimensions, three of which pertain to the conceptualization of what an anesthesiologist is, and three of which pertain to the approach to learning anesthesia: *Curriculum scope, Curriculum complexity, Curriculum verity and Learning resources, Learning responsibility, Learning verification*. The relationships between the three conceptions and the six dimensions of variation are represented in an outcome space (Table 4.) showing a hierarchy of increasing understanding.

**Table 4:** Outcome space

		Conceptions		
		**Learning competences of Anesthesia**	**Learning work of an Anesthesiologist**	**Learning being an Anaesthetic professional**
Dimensions	Curriculum scope	Formal curriculum (FC): *“seeing them* (answers) *in black and white makes me feel like I truly understand* Interview 2, line 32	FC + Informal curriculum (IC): *“gut feeling that guides all my choices. It’s the sum of previous experiences.”* Interview 1, line 60	FC + IC + Hidden curriculum: “*you still also have to learn the rules of that workplace* Interview 13, line 116
	Curriculum complexity	Isolated tasks: “*days, when I get to perform practical procedures… are educational* Interview 1, line 46	Combined tasks (CT): ” one *must also be skilled in the overall thinking and have the big picture and the global perspective, and it should work in parallel and with different types of thinking.“* Interview 14, line 71	CT + Dynamic & Context-specific: *“they* (educational supervisors) *get a better picture of you and how you react in different situations”* Interview 8, line 185
	Curriculum verity	Right and wrong: “*one believes someone who says something and then finds out later that it isn’t true” (a bad anaesthetist)* Interview 4, line 140	Personal judgements: “*So, it becomes like textbook knowledge, but still with someone’s experience.”* Interview 6, line 67	Judgements with uncertainty: “*difficult… to hold on to that still seems to have evidence for”* Interview 15, line 178
	Learning sources	Literature: “*I think about physiology and pharmacology and then I think about the guidelines”* Interview 10, line 58	Colleagues: “*good dialogue so that one can really discuss the handling together”* Interview 8, line 55	Non-hierarchical network: *”…work with nurse where there is a good dialogue so that you can really discuss the handling together with them. But it’s quite unpretentious.“* Interview 8, line 55
	Learning responsibility	Educational institution: “*during the 5 years, you are placed in different blocks”* Interview 4, line 61	Both educational institution & learner: *“being placed at different hospitals but taking it upon myself to develop…”* Interview 14, line 13	Mainly learner: “*if one had been completely passive, I don’t think one would have gotten there. It’s much about learning, making decisions oneself, and being able to stand by them.”* Interview 3, line 17
	Learning verification	Exams: “*I enjoy taking written exams because I like the visual confirmation of the answers”* Interview 2, line 32	Teachers: *“Above all from my supervisors and consultants”* Interview 11, line 23	Self-reflection: *“It’s not so that now one is finished. It’s a continuous development.”* Interview 15, line 112

Framework of conceptions & dimensions with respect to learning anesthesia in the context of a specialty training program

The following section presents an overview of the dimensions described in the study, followed by detailed descriptions of the conceptions, along with illustrative quotes from the interviews that highlight the various dimensions embedded within them. It is important to note that the aim of the study is not to categorize individual anesthesiologists but to analyze their collective experiences as recounted in their individual statements.

### Dimensions

Six dimensions were found as a result of the iterative analysis process.

#### Curriculum scope

There was variation in the depth and breadth of understanding of the true anesthesiology curriculum. The understanding ranged from only the formal competencies mentioned in the written curriculum to also including context-specific tacit knowledge, personal skills, and social skills from the hidden curriculum in the broadest view.

#### Curriculum complexity

The perceived level of complexity of the anesthesiology curriculum varied, with some perceiving it as simply a series of isolated skills, while the more advanced individuals viewed it as a complex, context-dependent, and dynamic model of combined skills.

#### Curriculum verity

Correct anesthesiology knowledge was understood differently. Some believed in a dualistic view where solutions can be learned from resources, while others saw it as context-dependent and uncertain.

#### Learning resources

Anesthesiologists had different approaches to acquiring anesthesiology knowledge. Some relied on textbooks and courses, while others depended on discussions with colleagues. Still, some sought to build a larger network that included both clinical and patient-related perspectives.

#### Learning responsibility

The study revealed variations in the responses regarding the perceived responsibility for learning anesthesia, ranging from the anesthesiologists themselves being responsible to the educational institution being responsible.

#### Learning verification

There was a difference in how individuals determined whether they had acquired the necessary knowledge to become an anesthesiologist. Some believed that verification came from passing exams, while others relied on feedback from educational supervisors. Meanwhile, some believed that verification could come from various sources that they needed to reflect on themselves.

### Conceptions

Three conceptions were found as a result of the iterative analysis process.

#### Learning competences of anesthesia

In the first concept, learning is focused on acquiring competencies from a formal curriculum with anesthesiology knowledge described in literature and taught through courses, with a focus on right and wrong approaches. The learning process is task-oriented, and there is an emphasis on separate, clear, quantifiable, and measurable requirements. The learning outcome is typically assessed through exams to measure the acquisition of knowledge and skills related to administering anesthesia to patients. In this process, the learner plays a passive role with the educational institution setting learning goals and providing learning opportunities. Interviewee quotes shown in Table 5.

**Table 5:** Learning Competences of Anesthesia: Interview excerpts

Dimension	Interviewee quote	Researcher comment
Curriculum scope	*“As someone who is at the beginning of my specialty training, there is a lot of emphasis on practical work right now. Days, when I get to perform practical procedures, such as epidurals or central lines, once or twice and then sit down and receive feedback, are very educational”* (Interview 1, line 45)	The main components of what is learned are given as specific procedures and detailed feedback in relation to those. No other levels of learning are indicated.
Curriculum complexity	“(An educational day) *is a day when I have had the opportunity to perform a practical procedure, either an intubation or a central venous catheter, once or twice.”* (Interview 1, line 93)	An educational day is described as a day where you learn a series of isolated practical skills.
Curriculum verity	*“Believing someone who says something and later finding out that it is not true, it’s easy to lose trust.“* (Interview 4, line 140)	There is a view that there is either a right or wrong way of performing anesthesia which should be known by the educational supervisor
Learning resources	*“It’s still necessary to take the time to read something, new concepts and so on, if one wants to learn something new. But there are also other things that one reads at work, such as routines and procedures”* (Interview 7, line 37)	The focus is learning from written literature.
Learning responsibility	*“If you are placed at this hospital, you will get different blocks. You start on one floor, such as the fourth floor, and then attend surgeries there. After that, you rotate through different blocks”* (Interview 4, line 10)	Anesthesiology training is understood as structured in terms of time and place, and individuals are guided through these structures by virtue of an educational program. The learner being a passive agent in this process.
Learning verification	*“I like taking exams, I enjoy the visual confirmation of what I know, and getting confirmation in black and white that I actually know something. So, I appreciated that the course ended with a rather difficult exam and that I did well on it. It felt like, well, then I don’t have to be so uncertain because I can at least know a little bit now.”* (Interview 2, line 32)	Exams are considered to be a reliable means of verifying the acquisition of anesthesiology knowledge.

#### Learning work as an anesthesiologist

In the second conception, the focus of learning is broadened beyond the acquisition of a formal curriculum. Instead, it includes developing a range of other skills and competencies, such as tacit knowledge which is an important part of communication, teamwork, leadership, and clinical decision-making. The learning process involves combining anesthesia skills within a clinical model structure. Correct knowledge is not only found in literature, but also formed by personal opinions based on clinical experiences. Therefore, it is understood that social interactions are required for achieving the desired learning outcomes. In this conception, there is a shared responsibility for learning between the learner and the educational institution. Interviewee quotes shown in Table 6.

**Table 6:** Learning Work as an Anesthesiologist: Interview excerpts

Dimension	Interviewee quote	Researcher comment
Curriculum scope	*“I have skied a lot and trained a lot of ski instructors, but I can’t really explain to anyone how to ski. You have to demonstrate, explain, and feel it. It’s something that somehow comes to you, and when you master it, it just exists within you.“* (Interview 15, line 115)	This person is trying to describe tacit knowledge in anesthesia by using learning to ski as an analogy.
Curriculum complexity	*“So, I have experienced some of my colleagues, at least, that they have developed and started seeing things in a different way. They think about other things and focus on other things. They are a little more flexible and not just stuck in their thoughts and ideas.”* (Interview 11, line 186)	There is an understanding that in anesthesia you require to have a broad and flexible view
Curriculum verity	*“It is easy for me to assume that something should be done a certain way just because I have read it in books, but if someone has extensive experience with it, I must respect their opinion.”* (Interview 3, line 160)	It is understood that anesthesiology knowledge is influenced by personal opinions.
Learning resources	*“I never feel like I am coming up with ideas on my own without discussing my thoughts with someone else. I go through what I am thinking of doing with a patient with either the nurse standing next to me, bounce some ideas off them, or with another doctor who has more knowledge than myself, see what is reasonable.“* (Interview 2, line 66)	This person appreciates the discussions had with educational supervisor as a learning source.
Learning verification	*“I do most of it, but we talk about how to do it and he guides me through it and such. Even better, afterwards we can sit down and discuss what I found difficult and he can give me feedback.”* (Interview 10, line 83)	An educational supervisor explains what constitutes correct anesthetic knowledge

#### Learning being an anesthesiology professional

This last conception highlights the importance of developing a range of competencies beyond skills of a formal and informal curriculum. It acknowledges that patient care and safety require a multidisciplinary approach and that ethical and professional behavior are crucial in this very much social as well as clinical context.

The learner is seen as being responsible for their own learning process, recognizing the uncertainty of knowledge and seeking out different perspectives to develop a holistic understanding that is flexible and adaptable to different contexts and situations. This requires ongoing dialogue with a range of people, including colleagues, patients, and relatives.

The educational institution has a role in facilitating this learning process by providing a supportive learning environment, but the learner takes full responsibility for own lifelong learning and ongoing professional development. Interviewee quotes shown in Table 7.

**Table 7:** Learning Being an Anesthesiology Professional: interview excerpts

Dimension	Interviewee quote	Researcher comment
Curriculum scope	“*Just because you work so much with other people’s feelings and input, intensive care nurses, junior nurses and colleagues from other specialties etc., you need to take in all their opinions but at the same time make it clear that the responsibility of how you perform the anesthesiology and if patient should be admitted to intensive care or not lie with you.”* (Interview 8, line 218)	This reflects the understanding of the knowledge required to do anesthesia is influenced by the social context of the hospital setting and requires an understanding of the hidden curriculum
Curriculum verity	*“It can be very difficult with evidence and very difficult to validate it, so you try to find something that you can stick to that still seems to have evidence for it. But that changes over time“* (Interview 15, line 177)	This concept shows that there is an uncertain aspect to consider when it comes to anesthesiology knowledge
Learning resources	*“So, I overheard this person discussing with other colleagues, had read extensively, had made a plan, assessed the patient, and spoken with relatives present and, in the end, nothing went wrong because everything was so well prepared”* (Interview 2, line 155)	There is an understanding that a large network of people, clinical and non-clinical are sources of knowledge.
Learning responsibility	*“And that if you have questions, you get quite a lot back. Sometimes people may not say anything, but then maybe I’ll ask myself. Then one can choose whether to take it in or not. So, it’s up to oneself but also up to the environment in a way.”* (Interview 6, line 224)	It shows that the learner is taking full responsibility to learn
Learning verification	*“When I feel that I have learned something, it’s because I have learned something medical. That’s what matters to me. That’s what I value as something positive. Then I feel like I have learned something. But I could have been somewhere else and learned something completely different. Maybe I learned something about myself or how to interact in a group or something like that. I find that much harder and more personal.“* (Interview 15, line 216)	This shows a high degree of self-reflection on what has been learnt.

## Discussion

### Summary of findings

In this phenomenographic study, which interviewed anesthesiology trainees and specialists, we identified three qualitatively different understandings of what an anesthesiologist is, and of the learning process involved in becoming one. This variety exists despite all participants having experienced identical specialty training programs at the same university hospital in Sweden.

This variation of understanding can be described within a hierarchical framework of three conceptions, which are further described in six dimensions that illustrate understanding from a basic to a more discerning view. These differences in understanding show that there are aspects of what and how to learn anesthesia that are apparent to some but not to all. These relatively invisible elements include the need for tacit knowledge, complexity thinking, socialization, and taking one’s initiative and responsibility for learning. In broad terms, there is a developmental direction in understanding that corresponds to experience, but it is not something that is clearly predictive.

### Contribution to previous findings

- Understanding of how to learn – the learning process.

William Perry (1970), a renowned educational psychologist, introduced the groundbreaking notion that students’ perceptions of learning evolve gradually throughout their educational journey, traversing a predictable sequence of epistemological growth [18]. This developmental progression in comprehending the acquisition of knowledge has since found empirical support in the field of healthcare. Notably, Keskitalo et al. [19] demonstrated disparities between healthcare facilitators and students in their conceptions of teaching and learning. Similarly, Stoffels et al. [20] reported divergent perspectives on clinical learning among various stakeholders involved in nursing undergraduate education. Our study also reveals variations in the conceptions regarding the learning process when it comes to anesthesiology training. These conceptions can be categorized into different levels ranging from a basic understanding to a more advanced one. However, in our study, the years of experience did not consistently predict the level of understanding.

b. Understanding of what to learn.

Prior research has demonstrated the presence of variability in the perception of the desired learning outcome from a specialty training program in anesthesia, specifically regarding what constitutes an excellent anesthesiologist and their responsibilities. Larsson et al.[21] identified four distinct conceptualizations of anesthesia work among anesthesiology trainees. Similarly, St. Pierre & Nyce [22] uncovered disparities in understanding between novice and expert anesthesia practitioners regarding the development of anesthesia expertise. Additionally, Klemola’s examination of the clinical behavior of anesthesiologist [23] shed light on the differences in orientations among practitioners, resulting in diverse types of clinical behavior, such as realistic orientation versus objectivistic orientation. Haber’s qualitative study, titled “Exploring anesthesiologists’ understanding of situational awareness,“ [24] further delved into the comprehension of anesthesiologists regarding this crucial aspect. Consistent with the literature mentioned, our study also found differences in understanding of what constitutes an excellent anesthetist and their work in relation to a specialty training program. Furthermore, we identified various aspects where these variations occurred, such as in understanding of curriculum scope, learning resources, and learning responsibility. Moreover, there was a progression of understanding in relation to these aspects.

### Implications for specialty training of anesthesia

The lack of transparency in the anesthesiology curriculum has significant implications for anesthesia trainees, educators, and training program organizers alike. Anesthesiology trainees can find it challenging to identify the essential knowledge and skills needed to become an anesthesiologist, leading to confusion and uncertainty, potentially contributing to a stressful and demotivating learning environment. It is of course essential for educational supervisors to possess a comprehensive understanding of the curriculum, but they also need to recognize the trainees’ level of comprehension to facilitate effective professional development. Failure to have a shared mental model for learning anesthesia can lead to conflicts between trainers and learners, as described by St. Pierre et al.[22].

Specialty training program organizers must also ensure that they consider the entire anesthesiology curriculum to avoid counterproductive educational practices. These practices can include, on the one hand, irrelevant learning activities and assessments, or on the other, focusing learning activities solely on a small part of the curriculum, such as only on the formal curriculum. Overview of the complete curriculum is critical in establishing a learning environment that promotes the acquisition of the formal as well as the informal, and hidden curriculum. Finally, when selecting trainees for the specialty training program, their personal attributes necessary to learn all aspects of the curriculum should be considered.

### Study limitations and future studies

While many aspects of learning anesthesia within specialty training programs have been explored, several unanswered questions remain. One crucial question is how varying degrees of comprehension of learning anesthesia among anesthesiologists ultimately impact the quality and safety of patient care. The assumption is that greater depth of understanding leads to greater anesthesiology expertise, but is this true? With respect to this, it is important to further explore not only the anesthesiologists’ perceptions of anesthesia but also the patients’ perceptions, as well as how anesthesiologists perceive their role and professional identity in relation to the patient, in order to develop a teaching program focused on the most important stakeholder - the patient. It is also important to investigate whether educational inputs can influence the different levels of understanding, and whether some of those inputs are already in place within the present educational structure.

Furthermore, while our study provides valuable insights into anesthesia education in this particular setting, further research is needed to determine the generalizability of these findings nationally and internationally. It would also be pertinent to consider how applicable these findings are to the education of other medical and surgical specialties.

## Conclusion

This study has identified and described various student perspectives on what it means to be an anesthesiologist, as well as the learning process involved in becoming one. These perspectives vary from fundamental to more sophisticated concepts, providing a more comprehensive and nuanced understanding of learning taking place in an anesthesiology specialty training program. This has potential future benefits for:

1. Organizers of specialty training programs: Deeper understanding of the essential elements can aid in the constructive development of future anesthesiology specialty training programs.
2. Educational supervisors: Descriptions of different student perspectives can assist teachers in supporting their students by addressing their individual needs and viewpoints.
3. Specialty trainees: Having transparent information about all of the training requirements and expectations can ease learning for anesthesiology trainees.

## Data Availability

The datasets generated and/or analyzed during the current study are not publicly available due to promised anonymity of the participants but are available from the corresponding author on reasonable request and with permission of the participants in question.
